# Factors Associated With Perceived Stress Among Dementia Caregivers With Poor Sleep

**DOI:** 10.1093/geroni/igaa057.1380

**Published:** 2020-12-16

**Authors:** Yeonsu Song, Anna Papazyan, Monica Kelly, Sarah Choi, Constance Fung, Susan McCurry, Cathy Alessi, Jennifer Martin

**Affiliations:** 1 University of California Los Angeles, Los Angeles, California, United States; 2 VA Greater Los Angeles Healthcare System, North Hills, California, United States; 3 University of Washington, Seattle, Washington, United States

## Abstract

Poor sleep among family caregivers of individuals with dementia is associated with higher levels of caregiver stress. Modifiable factors that increase risk of stress among caregivers with poor sleep are targets for intervention. This analysis aimed to identify factors associated with greater caregiver stress among caregivers with poor sleep. Baseline data from an ongoing trial of a dyadic sleep intervention program for individuals with dementia and their caregivers with poor sleep quality (defined by Pittsburgh Sleep Quality Index total score > 5; N=21 dyads; mean age 70.8± 11.1 for caregivers, 80.5± 8.3 for care-recipients) were analyzed. Caregiving factors included Zarit Burden Index (ZBI) and SF-12 Short Form Health Survey (SF-12v2). Care-recipient factors included Mini-Mental State Examination (MMSE) and Revised Memory and Behavior Problems Checklist (RMBPC). Stress was measured with the Perceived Stress Scale (PSS). Analyses included Pearson correlations and t-tests. Caring for multiple care-recipients (n=5: 24.8±2.7) was associated with higher (worse) PSS scores than caring for one care-recipient only (n=16: 19.6±3.7, p=0.011). Caregivers with higher PSS also had a significantly higher ZBI score (r=0.53, p=0.015), higher distress related to care-recipient behaviors on the RMBPC (r=0.57, p=0.009) and worse mental health on the SF-12v2 (r= -0.47, p=0.037). PSS was not associated with care-recipient MMSE. These findings suggest that caregivers with poor sleep who care for multiple care-recipients may be at higher risk of stress. This work also identified potential targets (e.g., caregiver burden, mental health, distress related to care-recipient behavior) for reducing stress in this population.

